# Perception of Women with Lived Experience of Domestic Violence and Abuse on the Involvement of the Dental Team in Supporting Adult Patients with Lived Experience of Domestic Abuse in England: A Pilot Study

**DOI:** 10.3390/ijerph18042024

**Published:** 2021-02-19

**Authors:** Omolade Femi-Ajao

**Affiliations:** Division of Dentistry, University of Manchester, Oxford Road, Manchester M13 9PL, UK; omolade.femi-ajao@manchester.ac.uk

**Keywords:** women, domestic abuse, domestic violence, dentist, dental team

## Abstract

While there is existing evidence highlighting the important roles of health care professionals and the health care system in supporting patients with lived experience of domestic violence and abuse (DVA), there is a dearth of knowledge on the perception of dental patients on the involvement of the dental team, as health care professionals, in supporting adult patients experiencing domestic abuse. Data were collected from 24 women withs lived experience of domestic abuse using mixed methodological approaches and analysed using relevant analytic techniques. Results from this pilot study show that patients want to be asked about their experience of domestic abuse. Although there were barriers to disclosing to the dentist, evidence from this pilot study highlights that the dental practice is an avenue that can be used for appropriately signposting patients to relevant services. Evidence from this pilot study contributes to the existing knowledge on the need to raise awareness among the patient population with lived experience of DVA that the dental team can support them. In addition, there is a need for on-going training to enhance the capacity of the dental team for appropriately supporting their adult patients with lived experience of DVA.

## 1. Introduction

Domestic violence and abuse (DVA) experienced by women is increasingly becoming a global health problem [1,2,3]. Historically, little attention has been paid to the negative impact of domestic violence and abuse [4,5]. However, with the World Health Assembly in 1996 declaring violence against women a public health issue [3], and the resultant increase in evidence from empirical research highlighting the public health impact of domestic violence on women, e.g., [2], global regulations and conventions have been enacted to end domestic violence and abuse against women and girls [6].

Developments in the field of women’s health over the last thirty years have also led to renewed interest in improving support for female victims and survivors, with many agencies and women’s rights activist groups highlighting the need for governments and health care providers to enhance disclosure practices among women with lived experience of abuse [1,7,8]. Therefore, in addition to domestic violence and abuse against women being identified as a violation of human rights [9,10], many of the published work has explored and situated the health impact of the abuse on women as the foremost negative impact of the violence and abuse [3,11,12]. Thus, health care professionals have been identified as being in strategic positions for identifying and supporting women, by aiding disclosure and help seeking [8,13,14].

### 1.1. Disclosure of Domestic Violence to Health Care Professionals

The National Institute for Health and Care Excellence [8] defined disclosure ‘as any occasion when an adult or child who has experienced or perpetrated domestic violence or abuse informs a health or social care worker or any other third party’ [8] (p. 39). While there is existing evidence to support that health care professionals may be in a position to help mitigate the short-term and long-term negative effect of domestic violence and abuse on women through early identification and intervention, especially when presenting with bruises and injuries in an emergency care setting [15,16], many of these women have been known to withhold information about their injuries [17]. 

While evidence suggests that some women were able to disclose experiences of domestic violence after being asked by health care professionals in a supportive manner [17], it has been reported that health care professionals may miss opportunities to support women, especially where there was a lack of sensitivity in how women presenting with injuries resulting from domestic violence and abuse were asked about their injuries [18,19]. Thus, conclusions from a systematic review of literature on screening for domestic violence by health care professionals [20,21,22] have highlighted the importance of training health care providers, to facilitate disclosure of domestic violence and abuse within the health care setting, as a necessary step towards identifying women who experience domestic violence in order to offer help and support.

### 1.2. The Present Study

This pilot study was conducted among women with lived experience of domestic violence and abuse, on their perception of the role of the dental team in supporting adults with lived experience of domestic abuse. Although the National Institute for Care Excellence (NICE) DVA guidelines recommend an active role for health care professionals in identifying, supporting and referring to specialist advocacy services people experiencing DVA [8], existing evidence suggests limited engagement of the dental team in appropriately supporting female patients experiencing DVA utilising NHS dental services [18,23]. 

The importance of the dental team in getting involved in supporting adult patients experiencing DVA has been highlighted [24,25], particularly as existing evidence has shown that most victims of physical domestic violence sustain dental injuries [26] and it has been estimated that 65–75% of violence and abuse involves trauma to the head, neck and facial structures [27,28]. While there have been some studies conducted among dental care professionals to enhance their capacity for appropriately supporting adult patients with lived experience of DVA [24,25,28], there is a dearth of evidence exploring the perception of patients with lived experience of DVA on whether their dental care team should ask them about their lived experience of domestic abuse. 

Hence, this present study is important, given existing evidence which suggests that patients with lived experience of DVA may not necessarily feel comfortable disclosing the truth about their injuries [29], despite the expertise of the dental team in dealing with such disclosures. It is expected that findings from this study will contribute to the knowledge-base on appropriate strategies for patient engagement in order to ensure the uptake and utilisation of services within dentistry for supporting adult patients with lived experience of DVA.

## 2. Methods

A mixture of qualitative and quantitative data collection approaches was used in this pilot study, which was based on a cross-sectional study design [30]. Cross-sectional study design has been shown to be cost effective and safe for use in studies exploring traumatic life events [31]. As operationalised in this pilot study, study participants were only contacted once to complete the study questionnaire. This ensured reduced opportunity for women to relive the trauma of their DVA experience, and it also resulted in practical use of staff members’ time, while facilitating the data collection process. The use of mixed methods in cross-sectional studies has been shown to enhance the data collection and process, with a subsequent impact on the analysis and interpretation of data generated [32]. While there are disadvantages of using a cross-sectional study design, considering that data are collected at one time point [30], it is a useful research design for conducting pilot studies, as evidence generated can serve as the basis for developing other research studies on the topic under consideration [33]. Furthermore, using mixed methods in data collection may provide further insight that might be missed if only one methodological approach was used in data collection [30].

### 2.1. Study Aim

The aim of this pilot study was to understand the perception of women with lived experience of domestic violence and abuse (DVA) on the involvement of the dental team in supporting adult patients with lived experience of DVA.

### 2.2. Recruitment and Data Collection

Study participants for this pilot study were recruited via a DVA professional service provider in North-West England. Data collection took place between May and July 2018. In accordance with established methodological and ethical approaches, surveys were used for data collection, and staff members within the DVA service facilitated the data collection process in order to protect the anonymity of study participants as well as provide any additional support that might be required, especially if study participants become distressed as a result of participating in this study. In total, 24 women participated in the pilot study. All study participants have a lived experience of domestic abuse, and 22 women (91.7%) were registered with a dentist at the time of the abuse, and at the time of data collection.

### 2.3. Data Analysis and Management

#### 2.3.1. Quantitative Data

Quantitative data were managed using IBM SPSS version 25 (IBM Corp., Armonk, NY, USA). Descriptive statistics were used to enhance data cleaning, analysis, and interpretation. Findings from the statistical analysis are presented in the results section. 

#### 2.3.2. Qualitative Data

Qualitative data were collected as responses to open-ended questions included in the survey. These were entered verbatim into IBM SPSS version 25 as individual survey responses to facilitate textual analysis of the responses. Qualitative analysis was conducted using the Framework approach [34] in order to enhance our understanding of quantitative data collected in this study. Hence, as a methodological approach, triangulation was not used in this study. 

Using evidence from Gale et al. [35], the six stages of Framework Analysis were implemented. However, due to the method used for data collection and the volume of data, some of the stages were combined. As operationalised in this study, stage 1 (transcription) and stage 2 (familiarisation with the interview) were combined, given that the qualitative data were collected as responses to the open-ended questions included in the survey. Stages 3 and 4 (coding and developing an analytical framework) were also combined to ensure that the codes developed informed the analytical framework to be used in understanding the emerging evidence from the qualitative data. Since this was a pilot study, a pragmatic and deductive approach was also adopted in applying the analytical framework and charting data into the framework matrix [35].

### 2.4. Characteristics of Study Participants 

All study participants were adults, over 18 years. As shown in Table 1 below, although most of the study participants were aged between 18 years and 39 years old (N = 19; 79.2%), the highest age groups were women between 35 and 39 years old (N = 6; 25%). As a combined group, women aged 40–65 years make up 20.8% of the study participants. The wide spread of the age of study participants highlights the importance of recognising that domestic violence and abuse affect women irrespective of age, with both younger and older women being affected [2].

Of the 24 study participants, a majority (58.3%) of study participants reported their ethnic origin as White British, followed by 25% of women reporting their ethnic origin as Asian, mainly Pakistani, and 12.3% of women indicating their ethnic origin as Black Caribbean/Mixed White and Black Caribbean. It is interesting to note that none of the study participants were from the Black African ethnic group or the other Asian (such as Chinese) ethnic group. While domestic violence services are available to all women irrespective of ethnic origin, existing evidence has shown that women who have insecure immigration in the UK are less likely to benefit from the range of services provided by professional DVA services [36]. As study participants were recruited from the population of women living in an accommodation managed by a DVA service provider, it could be inferred that the ethnic group of women in this study may reflect the ethnic origin of their usual service users. Thus, evidence from this pilot study corroborates existing evidence highlighting the lack of representation of ethnic minority and immigrant women in the utilisation of existing professional domestic abuse services [29,37].

## 3. Results

### 3.1. Acceptability of Questioning about Domestic Abuse

Although previous studies conducted among dentists on their involvement in asking patients about domestic abuse suggests that dentists were afraid of offending patients by asking about domestic abuse [38], emerging evidence from this study suggests that patients want to be asked about their experience of DVA, especially if they present with injuries from DVA. As shown in Table 2 below, 87.5% of respondents in this study indicate that questioning dental patients about domestic abuse is acceptable. This finding corroborates existing evidence [39,40] which shows that patients want to be asked about their experience of DVA within the health care setting. 

Although study participants perceived it as part of the duty of the dentists to refer women for additional support once they make a disclosure, there were concerns about the capability of the dentist to respond to DVA. Hence, study participants were supportive of training the dental team on DVA and how to respond. 

### 3.2. Barriers and Facilitators of Help Seeking

In this section, a narrative summary of the qualitative data is used in discussing the barriers and facilitators of help seeking. As described by Dixon-Woods et al. [41], the use of narrative summary in mixed-methods study provides an integrative approach for incorporating evidence from qualitative data into quantitative evidence synthesis.

Despite women agreeing that questioning about IPV is acceptable, when asked about previous disclosure, only one woman had previously disclosed to their dentist. A plausible explanation for this may be that women were not directly asked whether their dental trauma was as a result of DVA. As disclosed by study participants, 70.8% had not disclosed their lived experience of domestic abuse to their dentist (Table 3) despite the potential for being supported by their dental team [25]. 

In discussing the barriers to disclosure, evidence from the qualitative component of this study found that participant expressed concerns regarding the time available for consultation, the availability of other staff in the room, language barriers and the use of family members as interpreters, limited knowledge about the appropriateness of disclosing to the dentist, the patient’s own embarrassment and lack of confidence. These barriers have been reported as common issues with disclosing to staff within health care settings [42]. 

With respect to factors influencing disclosure, a study participant reported that:

*“My Dentist picked up on my problems as I was emotional on a visit last year. He handled it delicately (as did his nurse) he also handed me printed out information on police, victim support, domestic abuse agencies. Even though I denied I needed them (at that time), I did go on to explore more and use them. He saved me!”*

(**SP04**)

Based on the experience of the patient above, the awareness of the dental team with respect to handling domestic abuse and the overall health of the patient informed the decision to ask about domestic abuse. As reported by the patient, she did not disclose her experience; however, being given the information required to make an informed decision about seeking help was instrumental in her utilising the services. This evidence corroborates existing evidence that patients need to be asked about their experience and should be signposted to appropriate services [24]. While it is known that adult patients with lived experience of domestic abuse should be given the autonomy to make the decision about disclosure and help seeking for their abusive experience [15], evidence from this pilot study underscores the importance of awareness and willingness on the part of the health care professional in providing appropriate support (such as signposting to relevant services) to enhance the decision-making process.

## 4. Discussion

### 4.1. Trust and Confidentiality 

Based on evidence from this pilot study, it was observed that women trusted their dentist to act appropriately to support their overall health. This corroborates existing evidence on the importance of trust in disclosures of domestic abuse experience [42]. However, there were concerns about the safety of women, especially when the perpetrator may be registered at the same dental practice. There is also concern about other team members in the room when disclosure is made, thus highlighting the need to reiterate the relevance of a whole-team approach to handling DVA disclosure within dentistry. Given that the delivery of dental care will involve more than one dental professional in the room, patients need to be made aware that the dental team have a duty of care to ensure confidentiality of issues discussed within the consultation setting.

### 4.2. Patients’ Awareness and Knowledge 

As health care professionals, dentists have a duty of care to support the overall health of their patient [43]. As noted by the study participants, their lack of knowledge about whether they could disclose their DVA experience to the dentist was a hindrance to their help seeking for their DVA experience. While it is not commonly perceived as a likely setting for disclosing DVA, existing evidence has shown that the dental practice is a unique setting that may likely facilitate disclosure, especially as there are opportunities to get the patient on their own, without the partner, a family member, or the perpetrator [24,25].

Hence, it is crucial for dental practices to let patients know that they can disclose their experience in a confidential and safe way, without detriment to themselves or at risk of the perpetrator becoming aware.

### 4.3. Opportunity for Addressing DVA against Adult Patients Utilising Dental Services

Although existing evidence has identified series of barriers that may prevent the involvement of the dental team in supporting adult patients experiencing DVA [25,44], recent increases in the incidence of domestic violence and abuse, as well as the on-going health, social and economic impacts on individual victims, survivors, their families, the NHS, and the wider society make the involvement of the dental team in supporting people experiencing DVA now very important [8,24].

Hence, it is vital that patients are aware that their dentist and the dental team are concerned with their overall health, as part of the wider health care team [23]. While it is not the duty of the dental team to deal with the DVA issue, dentists and their team have a duty of care to respond appropriately to disclosures, by ensuring that patients disclosing a lived experience of DVA are equipped with relevant information required to enable them to make an informed decision about help seeking from appropriate service providers.

Unlike other health care professionals, where it may not be possible to get the patient on their own, especially if the perpetrator or a family member accompanies the patient to the appointment, the nature of dental consultation is such that there are opportunities for patients to be seen by themselves, thereby giving the patient and the dental team an opportunity to ask the patient about domestic abuse incidences if there are concerns. Additionally, as it is likely that patients may be seen twice within a 12 month period, the dental team have another opportunity to ask about domestic abuse, especially if a pattern is observed in the dental trauma the patient presents with.

### 4.4. Implication for Practice

Although an emerging shift in the demographics of dentists has been observed [45], many practicing dentists are White British and men [45], which may affect the willingness of participants to disclose. This is due to existing evidence which shows that there is a gender dynamics in disclosure and help seeking, with women reported as being more likely to disclose to female health care professionals [46,47]. Hence, it is essential to ensure that appropriate prevention initiatives are put in place to make dental practices safe for women to disclose. This may be the involvement of dental nurses and hygienists in health education, as well as providing a safe space for women to know that they can disclose to any member of the dental team. 

As DVA is not primarily a clinical problem requiring diagnosis of a clinically-qualified health care professional, all relevant staff comprising the dental team need to have access to DV training on how to identify and make a referral to a designated DV professional service. This could be incorporated into the CPD training days, alongside other training such as First Aid and Safeguarding. DV training is different from safeguarding training, as many patients presenting with injuries likely resulting from IPV may not necessarily be vulnerable adults requiring safeguarding. 

## 5. Limitations of This Study

There are two key limitations that should be considered when interpreting the findings of this study. Firstly, this is a pilot study; hence, the sample size is small, and the findings are not generalisable. While this study provides an insight into the perception of women about the role of the dental team in supporting women with lived experience of domestic abuse, the sample size is not diverse enough to account for varied perceptions, which may be influenced by factors such as, education, socioeconomic status, ethnic origin, and immigration status. In addition, given that patients pay a certain amount for NHS dental services in England, the perception of women with lived experience of DVA who do not have the financial resources to cover their dental services may not have been captured by this pilot study. Similarly, patients who could afford to privately cover the cost of their dental care may also have different experiences with their dental team. Secondly, this study was conducted among women with lived experience of domestic abuse living in an accommodation operated by a DVA service provider in North-West England, thereby excluding the views of men and other groups not supported by professional DVA service providers or living in a DVA accommodation.

## 6. Conclusions

This paper reports evidence from a pilot study conducted in North-West England, UK, with the aim of understanding the perception of women with lived experience of domestic violence and abuse (DVA) on the involvement of the dental team in supporting adult patients with lived experience of DVA. Data were collected from 24 women with lived experience of DVA, recruited via a domestic violence and abuse service provider.

Evidence from this study highlights the importance of questioning patients utilising dental services if presenting with dental injuries suspected to be from domestic abuse. 

This pilot study corroborates existing evidence on the importance of questioning patients, trust and confidentiality, the safety of women, and the need for the involvement of the dental team in responding appropriately to disclosures of domestic abuse. It was also observed that gender dynamics might impact the disclosure and help-seeking process, with ethnic minority and migrant women not being able to fully access existing services. Furthermore, evidence from this pilot study contributes to the existing knowledge on the need to raise awareness among the patient population that the dental team (as part of the wider health care provider) has a duty of care to support them. In addition, there is a need for on-going training to enhance the capacity of the dental team for appropriately supporting their adult patients with lived experience of DVA.

## Figures and Tables

**Table 1:** Characteristics of Study Participants.

**Age**	**Frequency**	**Percent**
18–24	5	20.8
25–29	4	16.7
30–34	4	16.7
35–39	6	25.0
40–44	1	4.2
45–49	1	4.2
55–59	2	8.3
60–64	1	4.2
Total	24	100.0
**Ethnic Origin**	**Frequency**	**Percent**
White English/British	14	58.3
Mixed (White and Black Caribbean)	1	4.2
Mixed (prefer not to say)	2	8.3
Mixed (White and Asian)	1	4.2
Black Caribbean	1	4.2
Pakistani Asian	5	20.8
Total	24	100.0
**Number of Years of Registration with Dental Practice**	**Frequency**	**Percent**
Less than 5 years	13	54.2
5–10 years	6	25.0
15+	2	8.3
Missing	3	12.5
Total	24	100.0
**Frequency of Attendance at Dental Appointment**	**Frequency**	**Percent**
Twice a year or more	12	50.0
Once a year	10	41.7
Missing	2	8.4
Total	24	100.0

**Table 2:** Perception of Women on the Acceptability of Questioning about DVA by Dentists.

Response	Frequency	Percent
Disagree	1	4.2
Neutral	1	4.2
Agree	12	50.0
Strongly agree	9	37.5
missing value	1	4.2
Total	24	100.0

**Table 3:** Showing Whether Previous Disclosures Were Made to the Dentist.

Response	Frequency	Percent
No	17	70.8
Yes	1	4.2
Missing value	4	16.7
Not applicable	2	8.3
Total	24	100.0

## Data Availability

The datasets generated during and/or analysed during the current study are not publicly available due to ethical approval restrictions but are available from the corresponding author on reasonable request.
